# Retrieval of Publications Addressing Shared Decision Making: An Evaluation of Full-Text Searches on Medical Journal Websites

**DOI:** 10.2196/resprot.3615

**Published:** 2015-04-07

**Authors:** Xavier Blanc, Tinh-Hai Collet, Reto Auer, Pablo Iriarte, Jan Krause, France Légaré, Jacques Cornuz, Carole Clair

**Affiliations:** ^1^Department of Ambulatory Care and Community MedicineUniversity of LausanneLausanneSwitzerland; ^2^Service of Endocrinology, Diabetes, and MetabolismUniversity Hospital of LausanneLausanneSwitzerland; ^3^Medicine Faculty LibraryUniversity Hospital of LausanneLausanneSwitzerland; ^4^Research Centre of the Centre Hospitalier Universitaire de QuébecDepartment of Family Medicine and Emergency MedicineUniversité LavalQuébec, QCCanada

**Keywords:** information storage and retrieval, systematic reviews, PubMed, text mining, full-text search, decision making, shared decision making

## Abstract

**Background:**

Full-text searches of articles increase the recall, defined by the proportion of relevant publications that are retrieved. However, this method is rarely used in medical research due to resource constraints. For the purpose of a systematic review of publications addressing shared decision making, a full-text search method was required to retrieve publications where shared decision making does not appear in the title or abstract.

**Objective:**

The objective of our study was to assess the efficiency and reliability of full-text searches in major medical journals for identifying shared decision making publications.

**Methods:**

A full-text search was performed on the websites of 15 high-impact journals in general internal medicine to look up publications of any type from 1996-2011 containing the phrase “shared decision making”. The search method was compared with a PubMed search of titles and abstracts only. The full-text search was further validated by requesting all publications from the same time period from the individual journal publishers and searching through the collected dataset.

**Results:**

The full-text search for “shared decision making” on journal websites identified 1286 publications in 15 journals compared to 119 through the PubMed search. The search within the publisher-provided publications of 6 journals identified 613 publications compared to 646 with the full-text search on the respective journal websites. The concordance rate was 94.3% between both full-text searches.

**Conclusions:**

Full-text searching on medical journal websites is an efficient and reliable way to identify relevant articles in the field of shared decision making for review or other purposes. It may be more widely used in biomedical research in other fields in the future, with the collaboration of publishers and journals toward open-access data.

## Introduction

Full-text searches of articles are known to increase the recall, also called sensitivity, and defined by the proportion of relevant publications that are retrieved [1-3]. Full-text search techniques have already shown many advantages for biomedical research [4], especially in genetic studies [5]. Biomedical search engines, such as PubMed, are essential in the everyday life of researchers and clinicians, and with the exponential growth of the scientific literature [6]. However, full-text searches are rarely used in the medical field, partly due to resource constraints.

In contrast to a PubMed search, a full-text search permits the identification of articles whose keywords appear not only in the title or abstract, but also in the main content (eg, discussion). Furthermore, a full-text search can help to retrieve publications without abstracts. Those articles, such as editorials and debates, have an impact on readers [7,8], and may pave the way for novel concepts, such as shared decision making (SDM). SDM has been defined as a process by which healthcare choices are made jointly by the physician and the patient [9]. It is increasingly advocated as a model of best practice for decision making in the medical encounter [10-12] by combining best evidence with patient values and preferences.

In a recent study [13], we performed a systematic review of publications addressing SDM. We wanted to measure the growth of the SDM concept that seemed to appear increasingly in editorials and article discussions in high-impact medical journals. However, no reliable data could support this assertion. We therefore needed a full-text search method to retrieve publications where SDM does not appear in the title or the abstract.

The aim of the present study was to assess the reliability and efficiency of full-text searches in major medical journals for identifying publications containing the phrase “shared decision making” and to compare the results with a traditional PubMed search. If reliable and efficient, this methodology may be used for retrieving publications for systematic reviews on topics other than SDM.

## Methods

We selected the 15 journals with the highest 5-year impact factors in 2010 in the “general and internal medicine” category from the ISI Web of Knowledge Journal Citation Reports [14]. Moreover, the eligible journals had to exist prior to or since 1996 and publish original articles, letters, and editorials.

To identify publications containing the phrase shared decision making, referred to as SDM publications, we built a search strategy combining the following 6 phrases (1) shared decision making, (2) informed decision making, (3) shared medical decision making, (4) informed medical decision making (5) informed and shared decision making, and (6) informed shared decision making. None of the terms exist as a Medical Subject Heading (MeSH) term.

All publications released between January 1996 and December 2011 were eligible, because the concept of SDM began to appear significantly in medical literature in the mid 1990s [15,16]. Moreover, electronic publications only became widely available on journal websites around 1995-1997 due to changes in the publishing framework that permitted the use of automated search engines [17].

Publications were retrieved through the full-text search function on the journals' websites, usually located on the “advanced search” web page or on the publisher website if not available. The search engine of each journal website is handled similarly to that of PubMed, with Boolean operators and filters. It demands no particular informatics competency, but some manpower is required as the operation has to be repeated on each journal website. We refer to this search method as website full-text search. We included publications of any type, with the exception of cover pages, tables of content, and indexes (ie, authors or keywords).

To assess the performance of the website full-text search, we compared it with a PubMed search using a similar strategy (ie, the same 6 SDM-related phrases in the 15 journals from 1996-2011). PubMed searches were limited to titles and abstracts since full texts are usually not directly available on the PubMed platform, but rather through links to the journal websites.

We next compared the type of publications retrieved through website full-text vs. PubMed searches. We categorized the publication type through a bibliometric analysis and then dichotomized the results between research and non-research publications. Research publications included interventional and observational studies, systematic reviews, guidelines, and consensus publications, whereas non-research publications included non-systematic reviews, editorials, comments, letters, book reviews, conference publications, and others.

In contrast to PubMed searches, the website full-text search method relied on journal or publisher websites whose search syntaxes were not explicit. To compare our results with a validation dataset, we contacted the editorial board of each selected journal to request authorization to obtain all published materials since 1996. After receiving their authorization, we collected published material in an electronic version to build a custom-designed database of full-text publications. We designed and launched an automated search script (Python Software, version 2.6, Python Software Foundation, Wolfeboro Falls, NH, USA). We used the same 6 SDM-related phrases for publication retrieval. This text retrieval method on a locally stored full-text corpus is referred to as downloaded full-text search [18]. We assessed the numbers of retrieved publications, and the reliability and concordance between the website and downloaded full-text searches.

## Results

**Figure 1:**
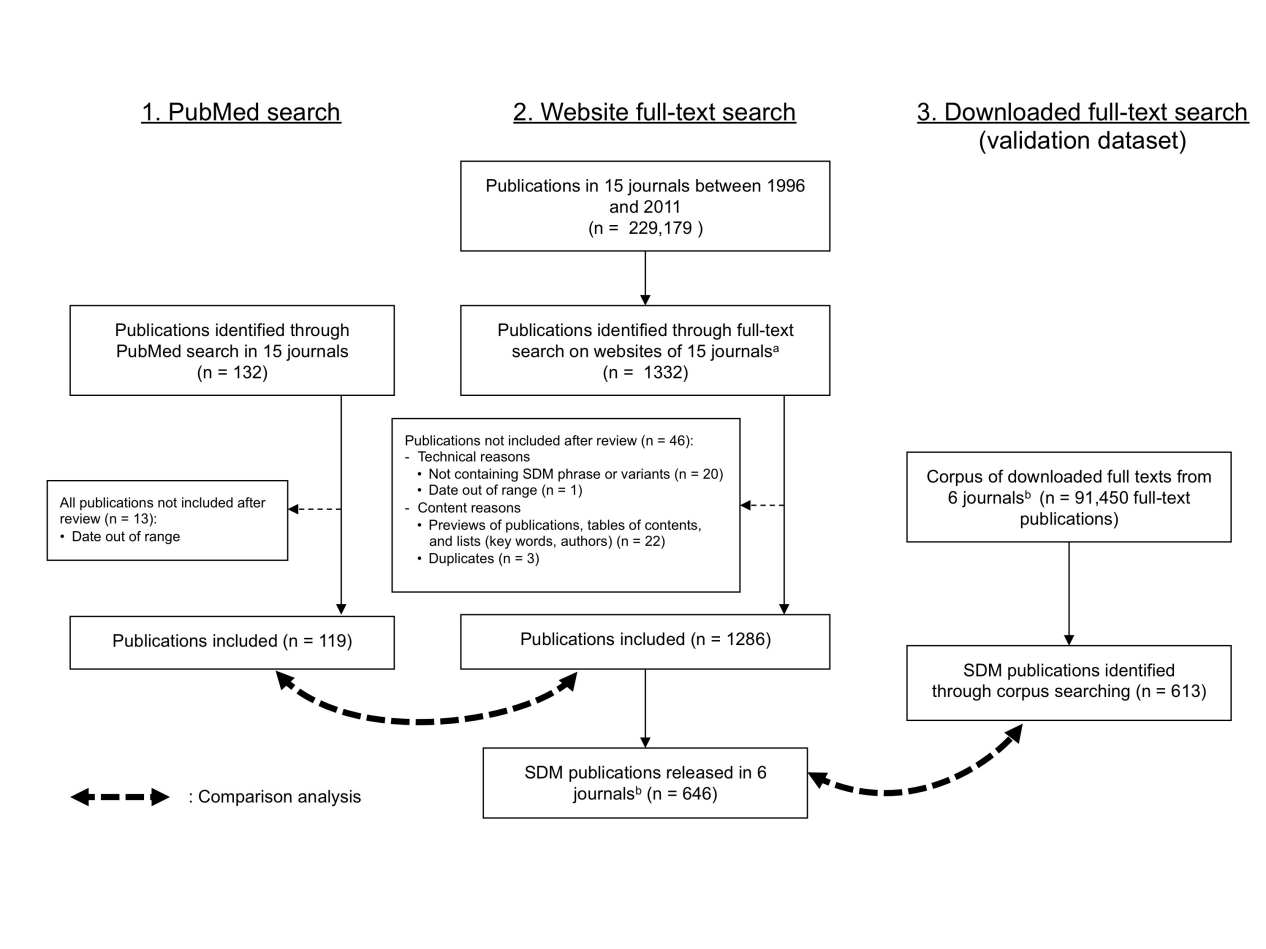
Flow chart of the search methods. ^a^Canadian Medical Association Journal : publications not available in full text for years 1996-1999, identified through PubMed Central. ^b^The 6 journals which collaborated for collecting full-text publications: British Medical Journal; Canadian Medical Association Journal ; Mayo Clinic Proceedings ; American Journal of Preventive Medicine ; Journal of General Internal Medicine ; Journal of Pain and Symptom Management.

Through the website full-text searches, we included 1286 SDM publications out of a total of 229,179 publications in the 15 journals from 1996-2011 (Figure 1).

**Figure 2:**
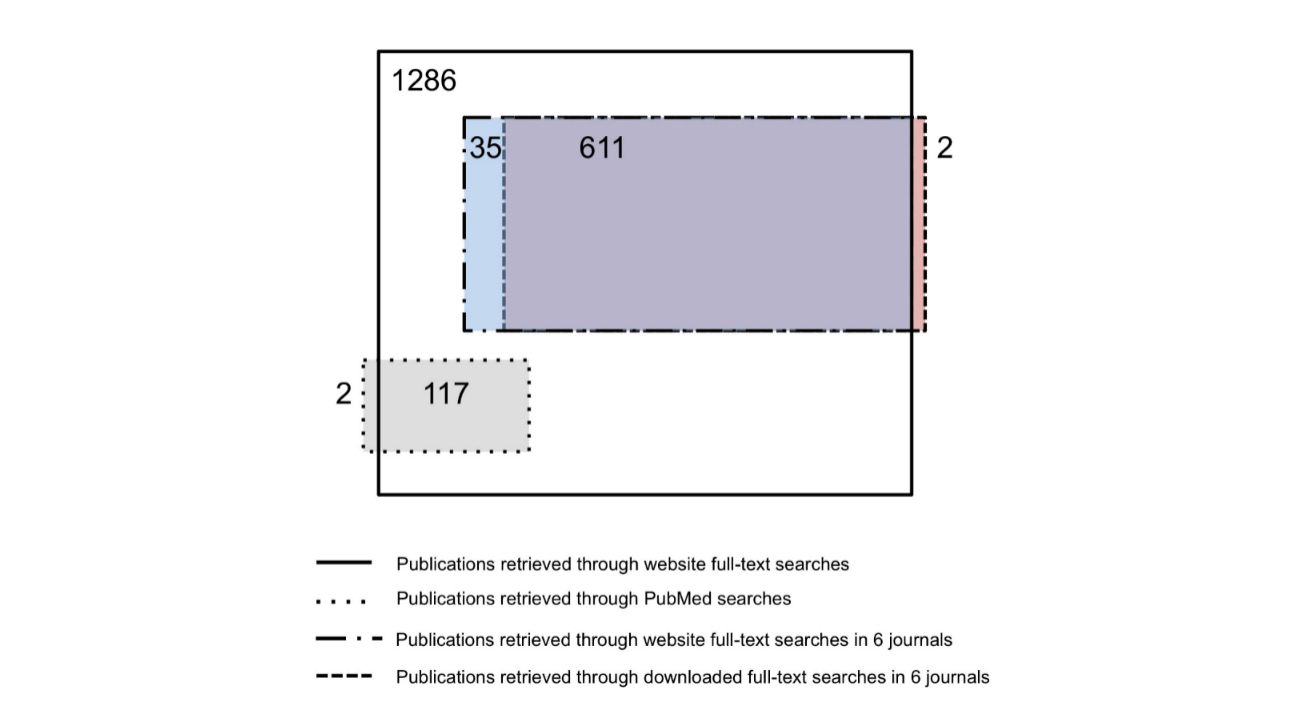
Adapted Venn schematic of the search method results. NB: Areas of the boxes are not exactly proportional.

Through the PubMed searches, 119 SDM publications were included. Of these publications, only 2 were missed by the website full-text searches; one due to unavailable data on the journal website for years 1996-1997, and while the other was available on the journal website, the browser failed to retrieve it (Figure 2). The BMJ published the highest number of SDM publications over 16 years with a minority found through PubMed searches (5.8%, 16/274) (Table 1). The Journal of General Internal Medicine and the JAMA followed with high numbers of SDM publications. Over time, the total number of SDM publications increased but the proportion found through the PubMed searches appeared to decrease from 11.5% (22/191) in 1996-1999 to 7.7% (39/505) in 2008-2011. A minority (36.3%, 467/1288) of all found SDM publications were research publications with the proportion of research publications higher through the PubMed search (52.1%, 62/119) compared to the website full-text search (36.2%, 465/1286). However, the PubMed search missed 86.7% (405/467) of the research publications containing the phrase shared decision making.

Of the 15 journals, 6 complied with our request to download all materials published during the study period. When limited to these 6 collaborating journals, 646 SDM publications were found through the website full-text searches, while the downloaded full-text searches retrieved 613 publications (Figure 1). When matching together the publications identified by both full-text searches, the concordance rate was 94.3% (611/648) (Figure 2). As well, 2 research article publications were retrieved by the downloaded full-text search, but not by the website full-text search. The reason for this was a defect in the automated Optical Character Recognition (OCR) of those publications.

**Table 1:** Characteristics of publications containing the shared decision making phrase according to the specific search method (N=1288).

Publication characteristics		Number of publications, n (%)			Total, n
		Found in PubMed only	Found in PubMed and website full-text search	Found in website full-text search only	
**Total publications**		2 (0.2)	117 (9.1)	1169 (90.7)	1288
**Journal**					
	British Medical Journal	0	16 (5.8)	258 (94.2)	274
	Journal of General Internal Medicine	0	37 (17.4)	176 (82.6)	213
	Journal of American Medical Association	0	15 (7.3)	191 (92.7)	206
	Annals of Internal Medicine	0	10 (8.5)	107 (91.5)	117
	Archives of Internal Medicine	0	10 (9.5)	95 (90.5)	105
	American Journal of Preventive Medicine	1 (1.2)	11 (13.3)	71 (85.5)	83
	Canadian Medical Association Journal	0	4 (5.6)	67 (94.4)	71
	The New England Journal of Medicine	1 (2.0)	1 (2.0)	47 (95.9)	49
	Journal of Pain and Symptom Management	0	1 (2.1)	47 (97.9)	48
	The Lancet	0	6 (14.6)	35 (85.4)	41
	Preventive Medicine	0	3 (10.3)	26 (89.7)	29
	The American Journal of Medicine	0	1 (3.6)	27 (96.4)	28
	Mayo Clinic Proceedings	0	2 (10.5)	17 (89.5)	19
	Journal of Internal Medicine	0	0	3 (100.0)	3
	Annals of Medicine	0	0	2 (100.0)	2
**Publication year**					
	1996-1999	2 (1.0)	20 (10.5)	169 (88.5)	191
	2000-2003	0	26 (9.2)	257 (90.8)	283
	2004-2007	0	32 (10.4)	277 (89.6)	309
	2008-2011	0	39 (7.7)	466 (92.3)	505
**Publication type**					
	^a^Research publications	2 (0.4)	60 (12.8)	405 (86.7)	467
	Non-research publications	0	57 (6.9)	764 (93.1)	821

^a^ Research publications are interventional and observational studies, systematic reviews, guidelines, and consensus publications.

## Discussion

### Principal Results

The website full-text searches identified 1286 SDM publications in 15 major medical journals, which was about 10 times more than through the corresponding PubMed searches. The search method was reliable with a good concordance rate when compared with a validation dataset of downloaded publications.

To our knowledge, full-text searches have never been assessed on medical journal websites. Our results are concordant with those of previous studies in other fields (eg, genomics), where the sensitivity of detecting keywords in full-text publications is much higher than when limited to a title and abstract PubMed search [19,20]. This may be especially useful when searching for information about study limitations or adverse drugs reactions [21], which are less likely to appear in abstracts or titles.

In a Cochrane review, handsearching identified more reports of randomized controlled trials than electronic searching through MEDLINE, particularly trials reported as letters, editorials, or journal supplements [22]. In the same way, our study showed that full-text searches found over 86.7% (405/467) of research publications that would have been missed through a simple PubMed search. The website full-text search method could be an efficient alternative to handsearching, where time and resources are limited.

The website full-text searches were fast and simple to perform compared to the downloaded full-text searches. While it took a few days to search the journal websites, it took 10-12 months of long negotiations to sign contracts with copyright holders to gain access to their material. However, we finally reached the goal for less than half of the contacted journals. Unfortunately, similar copyright issues have previously been reported in the text mining field [23].

### Limitations

Our study has some limitations. First, we did not develop a comprehensive search strategy, but selected only 6 phrases related to SDM. As a result, we did not assess the performance of an elaborate PubMed search strategy for SDM, compared with a full-text search method. We thought it was reasonable to explore this novel method with a simplified search strategy, as it is closer to the approach used by researchers and clinicians on a daily basis. Further studies should use a more comprehensive search strategy to compare extensively the new search method with PubMed or other search engines. Second, a gold standard search method could not be established, because it was not possible to verify that all publications with inclusion criteria were retrieved. For that matter, all three methods failed to identify all SDM publications, probably due to the lack of consistent indexing mechanisms and technical defects, like in the OCR. Third, for multiple resource constraints, we have not been able to perform the full-content analysis of the 1286 included publications. We are therefore unable to report on the meaning and the potential relevance of each publication. It is possible that some publications mentioned SDM just as a fashionable concept in a sentence or as a replacement term for other terms (ie, patient-centered care or risk communication). From this perspective and without a gold standard, we were unable to measure the proper recall and precision of the search method.

### Conclusions

Full-text searching of terms in medical journal websites is a reliable and efficient way to identify relevant articles in the field of SDM for review or other purposes. It may be more widely used in medical research in the future, with the collaboration of publishers and journals toward open-access data.

## Abbreviations

OCR: optical character recognition
SDM: shared decision making
